# Artificial intelligence based real-time prediction of imminent heart failure hospitalisation in patients undergoing non-invasive telemedicine

**DOI:** 10.3389/fcvm.2024.1457995

**Published:** 2024-09-20

**Authors:** Nils Hinrichs, Alexander Meyer, Kerstin Koehler, Thomas Kaas, Meike Hiddemann, Sebastian Spethmann, Felix Balzer, Carsten Eickhoff, Volkmar Falk, Gerhard Hindricks, Nikolaos Dagres, Friedrich Koehler

**Affiliations:** ^1^ Department of Cardiothoracic and Vascular Surgery, Deutsches Herzzentrum der Charité, Berlin, Germany; ^2^ Institute of Medical Informatics, Charité – Universitätsmedizin Berlin, Berlin, Germany; ^3^ Berlin Institute of Health, Charité – Universitätsmedizin Berlin, Berlin, Germany; ^4^ Berlin Institute for the Foundations of Learning and Data (BIFOLD), Technical University of Berlin, Berlin, Germany; ^5^ German Centre for Cardiovascular Research (DZHK), Partner Site Berlin, Berlin, Germany; ^6^ Centre for Cardiovascular Telemedicine, Deutsches Herzzentrum der Charité, Berlin, Germany; ^7^ Department of Cardiology, Angiology, and Intensive Care Medicine, Deutsches Herzzentrum der Charité, Berlin, Germany; ^8^ Institute for Bioinformatics and Medical Informatics, Eberhard-Karls-Universität Tübingen, Tübingen, Germany; ^9^ Department of Health Sciences and Technology, Translational Cardiovascular Technologies, Eidgenössische Technische Hochschule Zürich, Zürich, Switzerland

**Keywords:** heart failure, decision support (DS), telemedicine, machine learning, remote patient care, risk stratification

## Abstract

**Background:**

Remote patient management may improve prognosis in heart failure. Daily review of transmitted data for early recognition of patients at risk requires substantial resources that represent a major barrier to wide implementation. An automated analysis of incoming data for detection of risk for imminent events would allow focusing on patients requiring prompt medical intervention.

**Methods:**

We analysed data of the Telemedical Interventional Management in Heart Failure II (TIM-HF2) randomized trial that were collected during quarterly in-patient visits and daily transmissions from non-invasive monitoring devices. By application of machine learning, we developed and internally validated a risk score for heart failure hospitalisation within seven days following data transmission as estimate of short-term patient risk for adverse heart failure events. Score performance was assessed by the area under the receiver-operating characteristic (ROCAUC) and compared with a conventional algorithm, a heuristic rule set originally applied in the randomized trial.

**Results:**

The machine learning model significantly outperformed the conventional algorithm (ROCAUC 0.855 vs. 0.727, *p* < 0.001). On average, the machine learning risk score increased continuously in the three weeks preceding heart failure hospitalisations, indicating potential for early detection of risk. In a simulated one-year scenario, daily review of only the one third of patients with the highest machine learning risk score would have led to detection of 95% of HF hospitalisations occurring within the following seven days.

**Conclusions:**

A machine learning model allowed automated analysis of incoming remote monitoring data and reliable identification of patients at risk of heart failure hospitalisation requiring immediate medical intervention. This approach may significantly reduce the need for manual data review.

## Introduction

Heart failure (HF) is a major cause of mortality and morbidity and poses a substantial burden for the health care system. After the first HF related hospitalisation, the median survival is only 2.4 years (1), and every subsequent hospital admission further worsens prognosis (1). Furthermore, HF related hospitalisations are a main driver of health care related costs (2). Due to the ageing of the population and the increasing rate of comorbidities, HF related hospitalisations are expected to increase further. Thus, prevention of HF exacerbations requiring in-patient or emergency care management is a crucial aspect in HF management.

Early detection of patients with worsening HF status allows timely initiation of medical interventions that may prevent hospitalisations. In particular, this has been demonstrated in the setting of invasive hemodynamic monitoring by means of pulmonary pressure sensors (3). However, the invasive character of this monitoring combined with the high device costs are barriers to wide implementation (3).

As demonstrated in several randomised trials, remote monitoring of easily obtainable clinical parameters may also trigger early medical interventions and reduce the number of HF related hospitalisations and adverse events (4, 5). One of the major obstacles of broad application of remote monitoring of HF patients in clinical practice is the resource-intensive need for manual review of the collected data by trained personnel (6, 7). An automated assessment of incoming patient data regarding the risk for imminent events would allow focussing on patients most likely to benefit from medical contact and intervention, thus reducing the burden for health care professionals and allowing a much higher number of patients under such surveillance.

While numerous studies have made use of parameters routinely recorded as part of usual HF care to automatically assess patients’ health status (8–16), the literature on automatic assessment of frequently transmitted sensor data is much scarcer (17–24), especially in the context of validated, operational remote monitoring programs using non-invasive devices. Further, how automatic assessments could be effectively utilized within such a program, and how this affects the caregiver's patient capacity are currently open questions. With this work, we seek to lessen the gap in this research area by developing and validating a machine learning (ML) model for automated patient risk assessment by analysis of data from the Telemedical Interventional Management in Heart Failure II (TIM-HF2) randomised trial, and quantifying the resulting effect on the patient capacity through a simulation approach.

## Methods

### TIM-HF2 study design and participants

This analysis was conducted on the data of the TIM-HF2 trial, a randomised, multi-centre trial assessing the benefits of a structured remote patient management (RPM) programme. The design and main results of the trial have been reported previously (25, 26). In brief, TIM-HF2 was conducted in Germany between 2013 and 2018 and included 1,571 patients with a history of HF, New York Heart Association (NYHA) class II or III and a HF hospitalisation not longer than one year prior to randomisation, regardless of the left ventricular ejection fraction (LVEF). Patients were randomized to either RPM + usual care or to usual care only, and followed for 12 months. All patients underwent quarterly out-patient visits consisting of medical history, physical examination, collection of blood samples for biomarkers and assessment of concomitant treatments. Patients assigned to RPM were equipped with and trained in the use of a home telemonitoring system, which transmitted body weight, blood pressure, heart rate, ECG recording, peripheral capillary oxygen saturation, and self-rated well-being on a scale from one through five to a telemedical centre (TMC) on a daily basis. In the TMC, physicians and HF-nurses performed reviews of all patients’ incoming data and initiated interventions, if needed. The priority order for data review was defined by a pre-specified, conventional algorithm based on a set of heuristic rules (Supplementary Table 3) (25, 26).

For model development and validation in this current study, we used all patients of the full analysis set as defined in the TIM-HF2 trial. The final dataset contained 773 patients assigned to usual care, and 765 patients assigned to RPM + usual care.

All required ethics committee approvals, covering also the work presented here, were obtained.

### Outcome definition for the machine learning analysis

Primary outcome of the current analysis was unplanned HF hospitalisation occurring within seven days following data transmission.

### Candidate predictors

To account for the widely differing underlying baseline risk of the patients, we first created a new candidate predictor variable estimating the likelihood of all-cause death within one year based on variables that were gathered during the baseline out-patient visit prior to randomisation. For this purpose, 84 variables were considered (Table 1). The resulting predictor expressing the underlying baseline patient risk is hitherto referred to as *baseline risk variable*. The methodology for creation of the baseline risk variable was similar to the method applied for development of the main ML risk model and is described below.

**Table 1:** Baseline characteristics of the TIM-HF2 trial population, split 3:1 into training and validation set.

	Training set 1,153 patients	Validation set 385 patients
All-cause death within 12 months	112 (9.7)	38 (9.9)
Age (years)	73.0 [64.0, 78.0]	73.0 [65.0, 78.0]
Sex		
Female	339 (29.4)	129 (33.5)
Male	814 (70.6)	256 (66.5)
Weight (kg)	85.0 [74.0, 99.0]	84.0 [73.0, 98.0]
Body-mass index (kg/m^2^)	29.0 [25.2, 33.3]	28.1 [25.2, 33.4]
Days since last HF hospitalisation		
≤30 days	300 (26.0)	90 (23.4)
31–90 days	399 (34.6)	159 (41.3)
>90 days	454 (39.4)	136 (35.3)
Living alone	321 (27.8)	114 (29.6)
Living in rural area	678 (58.8)	237 (61.6)
Current or former smoker	551 (47.8)	173 (44.9)
Remote patient management	570 (49.4)	195 (50.6)
NYHA class		
II	582 (50.5)	186 (48.3)
III	571 (49.5)	199 (51.7)
LVEF	40.0 [30.0, 50.0]	41.0 [30.0, 51.0]
Heart rate (1/min)	71.0 [62.0, 80.0]	70.0 [61.0, 81.0]
Blood pressure (mm Hg)		
Systolic	123.0 [110.0, 140.0]	125.0 [110.0, 140.0]
Diastolic	74.0 [65.0, 80.0]	72.0 [65.0, 80.0]
Laboratory data		
GFR (ml/min per 1.73 sqm body surface area)	61.8 [44.4, 88.6]	63.0 [46.5, 86.1]
Haemoglobin (g/dl)	13.2 [12.1, 14.3]	13.4 [12.1, 14.5]
Hematocrit (%)	40.0 [37.0, 43.0]	40.0 [37.0, 43.0]
Leukocytes (1/µl)	7,600.0 [6,390.0, 9,100.0]	7,860.0 [6,427.5, 9,200.0]
Thrombocytes (1/nl)	209.5 [172.0, 250.0]	203.0 [164.2, 248.8]
Creatinine (mg/dl)	1.2 [1.0, 1.7]	1.2 [1.0, 1.6]
Sodium (mmol/L)	140.0 [137.0, 142.0]	140.0 [138.0, 142.0]
Potassium (mmol/L)	4.5 [4.2, 4.9]	4.5 [4.2, 4.9]
NT-proBNP (pg/ml)	1,438.5 [603.0, 3,223.8]	1,402.0 [628.4, 2,658.0]
MR-proADM (nmol/L)	1.1 [0.8, 1.5]	1.0 [0.8, 1.4]
MR-proANP (pmol/L)	257.9 [162.6, 389.2]	252.0 [166.4, 371.6]
Procalcitonin (µg/ml)	90.0 [70.0, 120.0]	90.0 [70.0, 120.0]
Pre-existing conditions		
Coronary heart disease	664 (57.6)	229 (59.5)
Inflammatory heart disease	47 (4.1)	15 (3.9)
Myocardial infarction	324 (28.1)	95 (24.7)
Dilated cardiomyopathy	376 (32.6)	115 (29.9)
Arterial hypertension	934 (81.0)e	308 (80.0)
Heart valve disease	584 (50.7)	204 (53.0)
Hyperlipidemia	626 (54.3)	207 (53.8)
Kidney failure	584 (50.7)	197 (51.2)
Peripheral artery disease	123 (10.7)	42 (10.9)
Stroke	125 (10.8)	40 (10.4)
Hyperthyroidism	37 (3.2)	15 (3.9)
Hypothyroidism	121 (10.5)	46 (11.9)
Malignoma	80 (6.9)	26 (6.8)
Liver cirrhosis	20 (1.7)	13 (3.4)
Coronary revascularisation	421 (36.5)	139 (36.1)
Bypass surgery	203 (17.6)	76 (19.7)
Heart valve surgery	111 (9.6)	46 (11.9)
TAVR	39 (3.4)	14 (3.6)
Mitra clip	47 (4.1)	13 (3.4)
Ablation of pulmonary veins	85 (7.4)	38 (9.9)
Pacemaker		
Single chamber	49 (4.2)	17 (4.4)
Dual chamber	116 (10.1)	26 (6.8)
Cardiac resynchronisation therapy	184 (16.0)	56 (14.5)
Implantable cardioverter defibrillator	354 (30.7)	102 (26.5)
Diabetes mellitus	522 (45.3)	180 (46.8)
COPD	207 (18.0)	67 (17.4)
Dyspnea		
On exertion	1,054 (91.4)	349 (90.6)
While resting	55 (4.8)	20 (5.2)
Peripheral edema	430 (37.3)	129 (33.5)
Cervical vein congestion	117 (10.1)	47 (12.2)
Pulmonary rattling noise	58 (5.0)	22 (5.7)
Pacemaker rhythm	312 (27.1)	90 (23.4)
Atrial fibrillation	405 (35.1)	142 (36.9)
AV block		
I	131 (11.4)	37 (9.6)
II	4 (0.3)	2 (0.5)
III	10 (0.9)	5 (1.3)
Left bundle branch block	276 (23.9)	75 (19.5)
Concomitant treatment		
Limited fluid intake		
≤2 L/day	190 (16.5)	89 (23.1)
≤1.5 L/day	531 (46.1)	158 (41.0)
≤1 L/day	8 (0.7)	3 (0.8)
ACEI	569 (49.3)	206 (53.5)
Statin	657 (57.0)	233 (60.5)
Allopurinol	221 (19.2)	68 (17.7)
*β*-blockers	1,048 (90.9)	348 (90.4)
ARB	408 (35.4)	127 (33.0)
Aldo blockers	604 (52.4)	188 (48.8)
Diuretics	1,106 (95.9)	366 (95.1)
Antiplatelet therapy	478 (41.5)	153 (39.7)
Anticoagulants	686 (59.5)	257 (66.8)
Calcium antagonists	242 (21.0)	90 (23.4)
Digitalis glycosides	182 (15.8)	58 (15.1)
Antiarrhythmic drugs	143 (12.4)	59 (15.3)
Nitrates	47 (4.1)	22 (5.7)
Ivabradine	37 (3.2)	17 (4.4)
Insulin	257 (22.3)	90 (23.4)
Oral antidiabetics	287 (24.9)	90 (23.4)

Data are median [lower quartile, upper quartile], or *n* (%). NYHA, New York Heart Association; LVEF, Left ventricular ejection fraction; GFR, Glomerular filtration rate; TAVR, Transcatheter aortic valve replacement; COPD, Chronic obstructive pulmonary disease; ACEI, Angiotensin-converting-enzyme inhibitors; ARB, Angiotensin receptor blockers; HF, Heart failure; NTproBNP, N-terminal prohormone brain natriuretic peptide; MR-proADM, Mid-regional proadrenomedullin.

For development of the main ML model for prediction of unplanned HF hospitalisation within 7 days following data transmission, we considered 18 variables (5 binary, 13 numerical) resulting from daily data transmissions, including ECG characteristics, blood pressure, oxygen saturation, weight, and self-rated well-being (Table 2). Additionally, we considered the baseline risk variable, and whether a previous hospitalisation due to HF occurred within 30 days prior to data transmission. This resulted in a total of 20 candidate predictors that were considered for the model.

**Table 2:** Characteristics of the RPM population of the TIM-HF2 trial going into the main ML model.

	Training set 183,070 individual patient data transmissions from 570 patients corresponding to 501.6 patient years	Validation set 61,640 individual patient data transmissions from 195 patients corresponding to 168.9 patient years
Days labelled as Hospitalisation due to HF within 7 days	1,043 (0.6)	425 (0.7)
Baseline risk score	0.2 [0.1, 0.3]	0.2 [0.1, 0.3]
Days labelled as HF hospitalisation discharge within previous 30 days	4,205 (2.3)	1,526 (2.5)
Self-rated well-being (1 [very good]–5 [very bad])	2.0 [2.0, 3.0]	2.0 [2.0, 3.0]
Weight (kg)	85.2 [74.3, 98.8]	86.4 [73.0, 102.5]
SpO2 (%)	96.3 [94.9, 97.6]	96.3 [94.9, 97.6]
Ventricular tachycardia events	343 (0.2)	145 (0.2)
Heart rate during ECG (1/min)		
Minimum	64.0 [57.0, 71.0]	64.0 [57.0, 72.0]
Maximum	76.0 [66.0, 87.0]	76.0 [67.0, 87.0]
Average	69.0 [61.0, 78.0]	70.0 [62.0, 79.0]
Blood pressure (mm Hg)		
Systolic	122.0 [110.0, 135.0]	123.0 [111.0, 136.0]
Diastolic	71.0 [64.0, 80.0]	72.0 [64.0, 81.0]
Average	97.0 [87.0, 108.0]	98.0 [88.0, 108.0]
Atrial fibrillation	61,501 (33.6)	23,191 (37.6)
AV block		
I	26,561 (14.5)	8,035 (13.0)
II	100 (0.1)	15 (0.0)
III	20 (0.0)	3 (0.0)
PQ Interval (ms)	174.0 [148.0, 207.0]	172.0 [148.0, 203.0]
QRS Interval (ms)	113.0 [96.0, 145.0]	111.0 [94.0, 141.0]
QT Interval (ms)	420.0 [389.0, 453.0]	414.0 [381.0, 449.0]
QT Interval, Bazett formula (ms)	450.0 [422.0, 481.0]	447.0 [421.0, 481.0]

The split into training and validation set is derived from the 3:1 split of the entire TIM-HF2 population described above. The dataset for modelling the risk of imminent HF hospitalisation is longitudinal. Each observation represents one day of transmitted data from one patient. Data are aggregated to median [lower quartile, upper quartile], or *n* (%) across all transmissions. HF, Heart failure; SpO2, Peripheral capillary oxygen saturation.

### Missing values

Missing values, or values set as missing because they exceeded plausible limits (Supplementary Tables 1 and 2) were scarce throughout the dataset. Biomarkers, which were collected quarterly during the study, contained up to 11.4% missing values. All other predictors contained less than 5% missing values. For daily transmitted variables, we used forward-filling to impute missing values where previous recordings were available, and backward-filling otherwise. For variables contained in the baseline risk model, we used linear regression imputation using up to four regressors chosen based on highest correlation.

### Model development

Model development and validation were performed in accordance with the guidelines for transparent reporting of a multivariable prediction model for individual prediction or diagnosis (TRIPOD) (27).

We performed a randomised 3:1 split of patients into a training set for model development, and a hold-out validation set. The split was stratified for all-cause death within one year.

For development of the baseline risk variable, we used a Random Forest classifier (28) trained on 1,153 patients from both the RPM and the usual care group. The effect of RPM was captured in a binary predictor.

**Figure 1:**
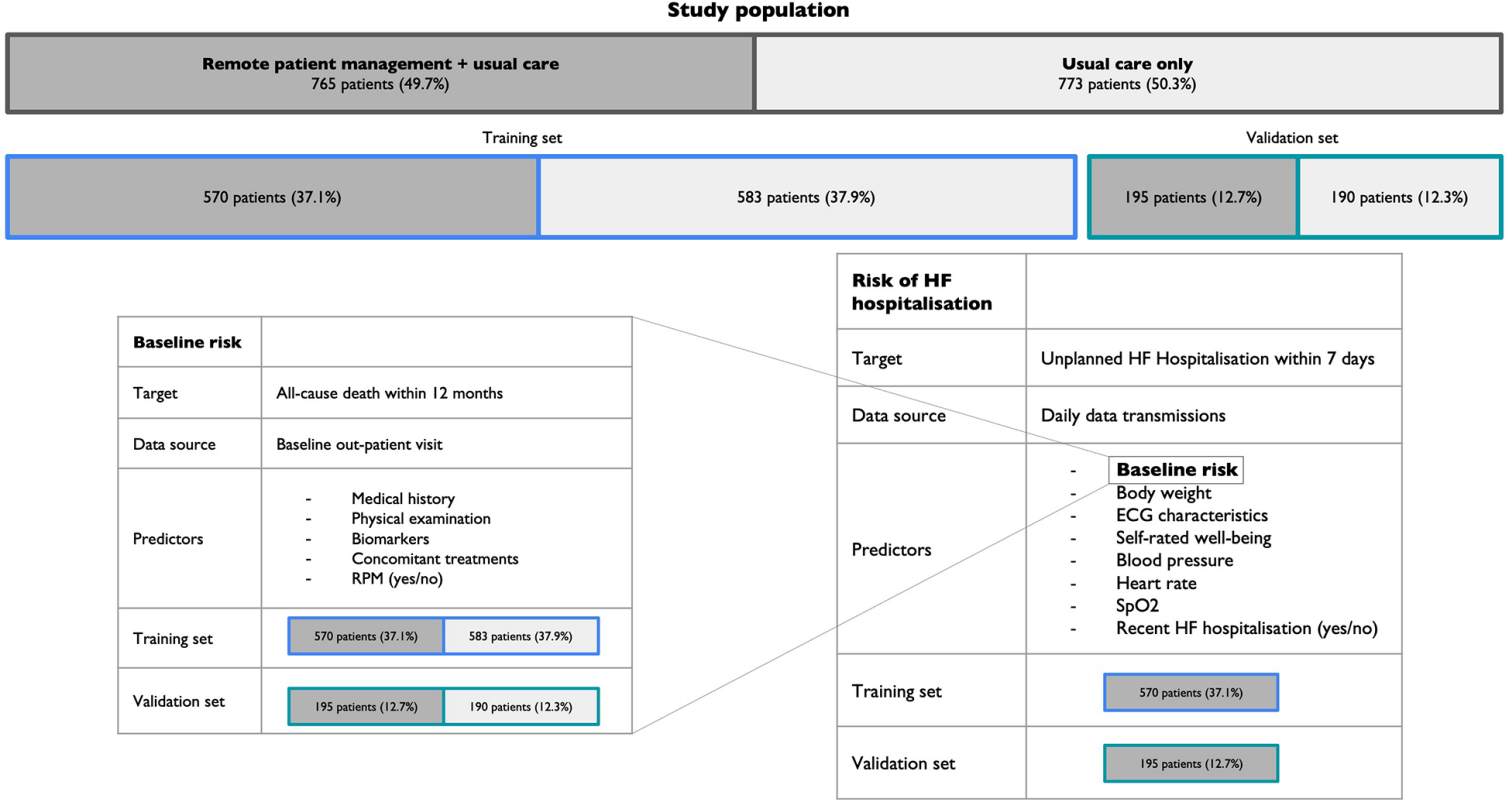
Model development and validation strategy. RPM, Remote patient management; HF, Heart failure; SpO2, Peripheral capillary oxygen saturation.

The risk for HF hospitalisation within the 7 days following data transmission was modelled via a Multilayer Perceptron (MLP) (29) using the daily data transmissions of 570 patients from the RPM group over a one-year study period. A summary of the model development and validation strategy is displayed in Figure 1.

We defined a space of potential model hyperparameters, over which we performed a randomised search (30) and chose the optimal values based on the highest area under the receiver operating characteristic (ROCAUC) in fold-wise cross-validation using only patients from the training set. With the optimal set of hyperparameters, we trained the final model on the entire training population. Implementation details are given in the Supplementary Material.

We initially included all available predictors and ranked them via repeated permutation importance (28) according to their univariate impact on ROCAUC. For development of the baseline risk variable, we eliminated an increasing number of low-ranked predictors, performed hyperparameter tuning using only the remaining ones, and settled for the combination of predictors yielding the highest cross-validated ROCAUC. For the main model with 20 initial predictors, we also calculated the permutation importance of each feature and excluded those with a non-positive importance.

All analyses were performed using Python, version 3.7 (31), specifically (but not exclusively) the scikit-learn package, version 0.24.1 (32) for building the ML pipeline, and TensorFlow, version 2.4.1 (33) for deep learning.

### Model validation

The model for the baseline risk variable was validated in the hold-out validation set of 385 patients. For reference, its performance was compared to the established Seattle Heart Failure Model (8).

The main ML model for prediction of unplanned HF hospitalisation within the following seven days was validated in the 195 RPM patients from the hold-out validation set. The performance of the model was compared with the performance of the conventional algorithm that was applied in the TIM-HF2 trial and that was based on a set of heuristic prioritisation rules (Supplementary Table 3) (26).

For visual inspection of discriminatory performance, we plotted the receiver operating characteristic (ROC) and precision recall (PR) curves. The PR curve displays the relationship between the sensitivity (also referred to as recall) and the precision (also referred to as positive predictive value), and can be informative in imbalanced classification problems, where the ROC can appear overly optimistic (34). For both ROC and PR, we additionally calculated the area under the curve (AUC). The 95% confidence intervals for the AUCs, and *p*-values for AUC comparisons were constructed using 10,000 bootstrap samples (35, 36).

We further visualized the trend of the score that was provided by the model in the 60 days preceding unplanned HF hospitalisations.

### Simulation of daily ranking

We assessed the feasibility of a policy focusing on daily review of transmitted data from high-risk patients only. For this purpose, a one-year long telehealth setting containing all 195 RPM patients from the validation set was emulated. The start date of RPM for all patients was artificially shifted to the same day, and patients were then continuously ranked on a daily basis based on their estimated risk for unplanned HF hospitalisation within seven days. We could thus estimate the proportion of HF hospitalisations within seven days following data transmission that would have been detected on a given day if only a certain fixed fraction of top-ranked patients had been clinically evaluated by the TMC staff.

## Results

### Baseline risk variable

**Figure 2:**
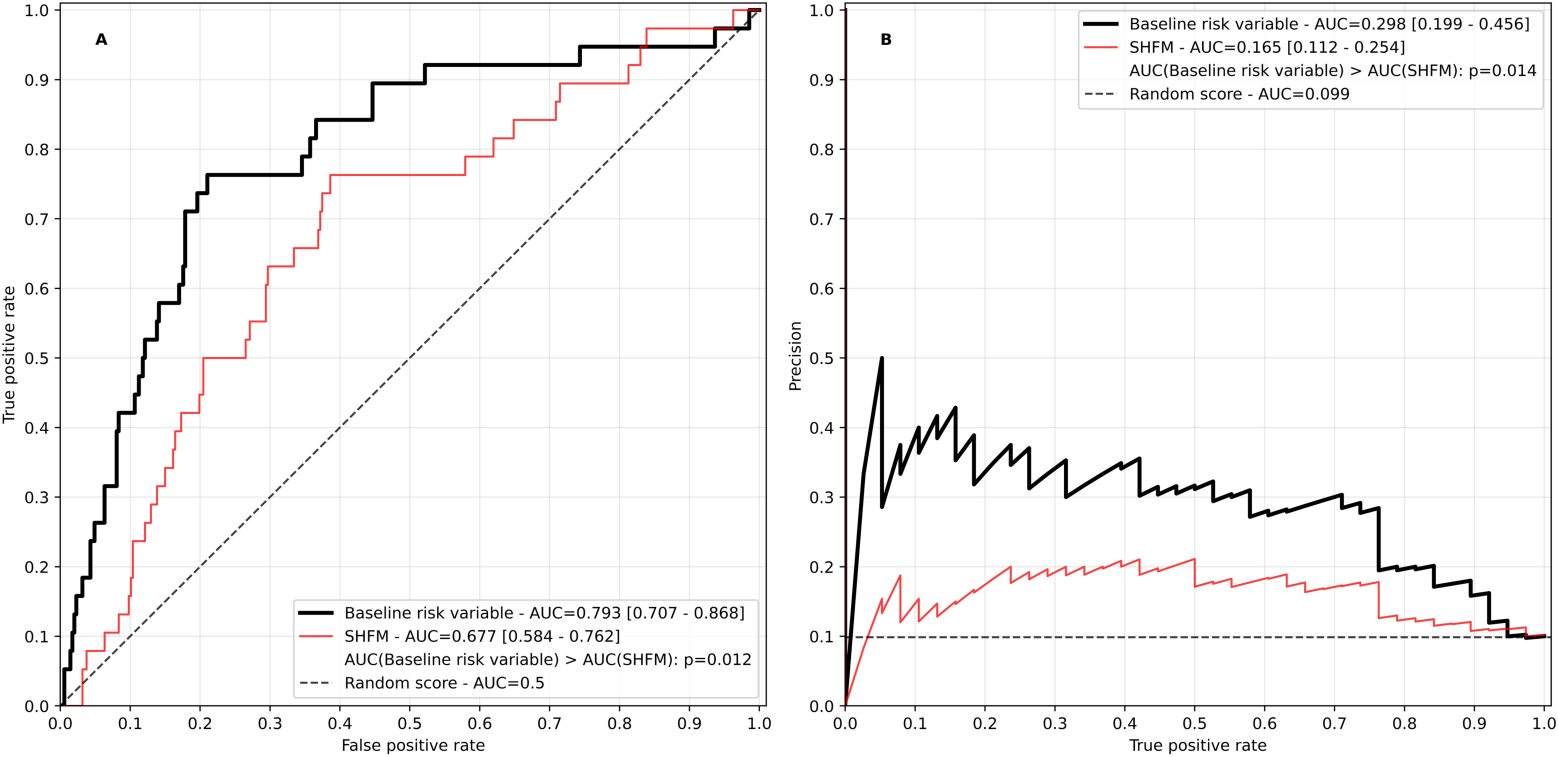
Receiver operating characteristic **(A)** and precision recall curve **(B)** of the baseline risk variable. The Seattle heart failure model (SHFM) score is plotted for reference. The SHFM score was calculated excluding information on uric acid, lymphocytes and cholesterol due to unavailability. AUC, area under the curve. Values in square brackets indicate the 95% confidence interval.

For the baseline risk variable, the predictor elimination process resulted in a final model of 27 predictors (Supplementary Table 4), of which N-terminal prohormone brain natriuretic peptide (NT-proBNP) stood out as the most impactful (Supplementary Figure 1). ROC and precision recall curves for the baseline risk variable and the SHFM are displayed in Figure 2. The baseline risk score had an overall satisfactory performance significantly outperforming the SHFM (AUC in ROC 0.793 vs. 0.677, *p* = 0.012; AUC in PR 0.298 vs. 0.165, *p* = 0.014).

### Prediction of unplanned HF hospitalisation within 7 days following data transmission

For the main ML model for prediction of unplanned HF hospitalisation occurring within the seven following days, the predictor elimination process resulted in a final model of 14 predictors (Supplementary Table 4). Among these, the most impactful was the baseline risk variable (Figure 6).

**Figure 3:**
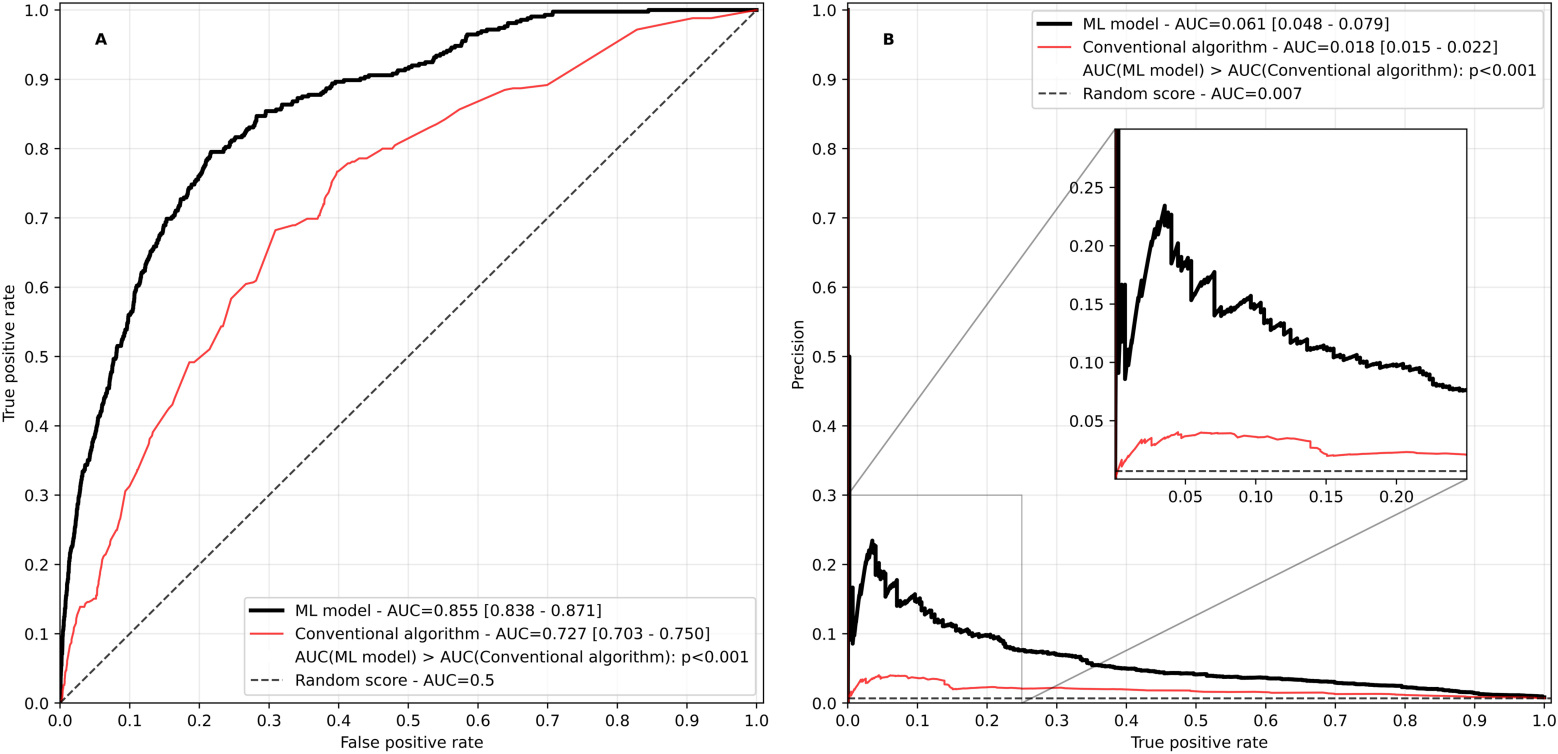
Receiver operating characteristic **(A)** and precision recall curve **(B)** of the ML-based risk score in comparison with the conventional algorithm based on heuristic rules used in the TIM-HF2 trial. AUC, area under the curve. Values in square brackets indicate the 95% confidence interval.

The model had a good performance with a ROCAUC of 0.855 and a PRAUC of 0.061, significantly outperforming the conventional algorithm based on a heuristic rule set that was used in the TIM-HF2 trial (ROCAUC 0.727, and PRAUC 0.018, *p* < 0.001 for both comparisons, Figure 3).

**Figure 4:**
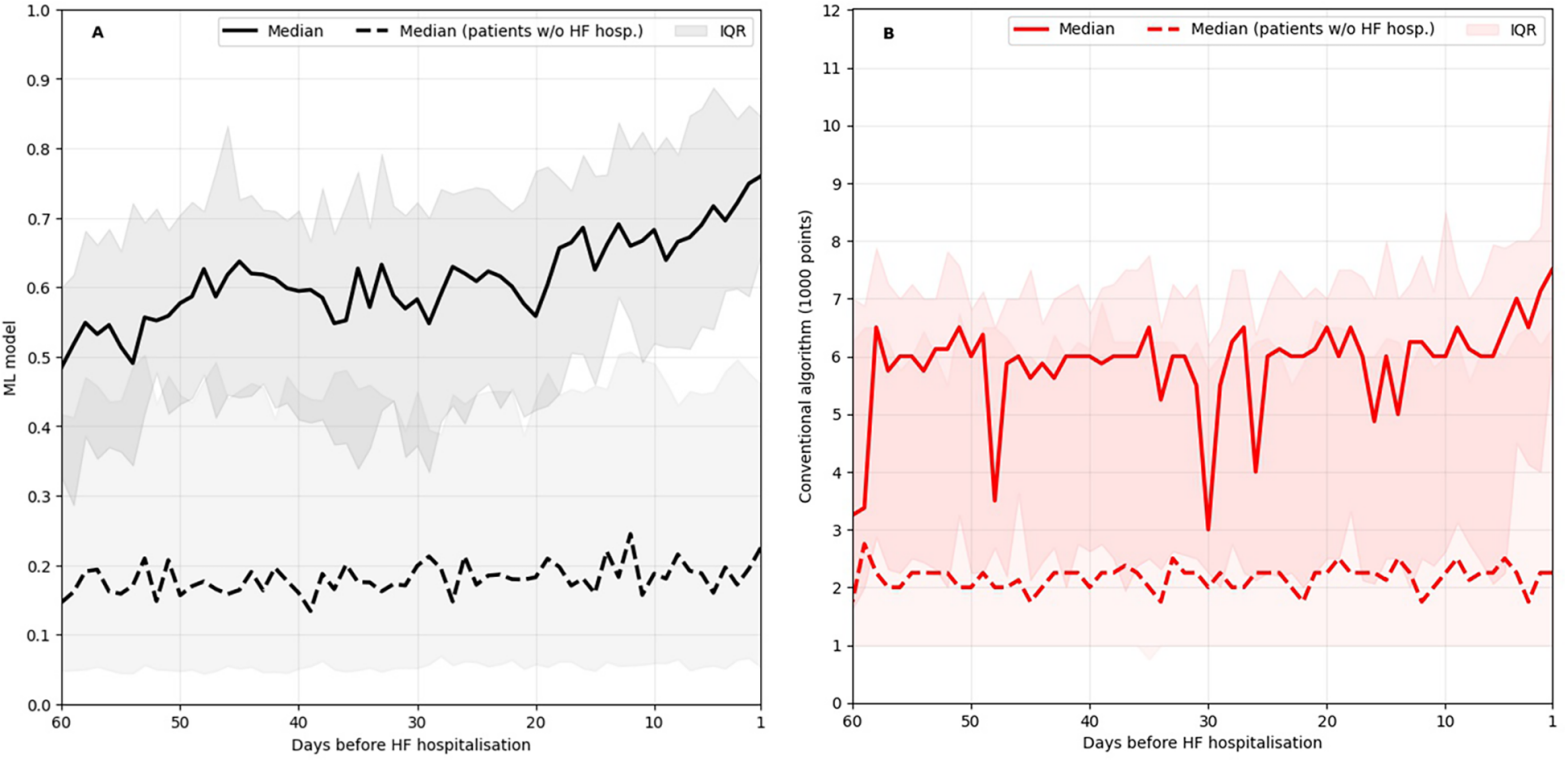
ML-based score evolution **(A)** and conventional algorithm evolution **(B)** leading up to HF hospitalisations in comparison to the evolution leading up to a random date for patients without HF hospitalisation. IQR, inter quartile range.

Figure 4 shows how the ML based model and the conventional algorithm evolved on average in the 60 days prior to an unplanned HF hospitalisation. The median score of patients in stable condition without HF hospitalisation approaching a randomly selected date within their follow-up period is shown for comparison. The median conventional algorithm score is highly volatile, and throughout the observed 60-day window, the median score of patients in stable condition is at times within the interquartile range of the score of patients approaching an unplanned HF hospitalisation. In contrast, the median ML-based score of patients approaching an unplanned HF hospitalisation is clearly separated from the score of patients in stable condition throughout the observed time window and exhibits a continuous upward trajectory starting approximately three weeks prior to a HF hospitalisation.

**Figure 5:**
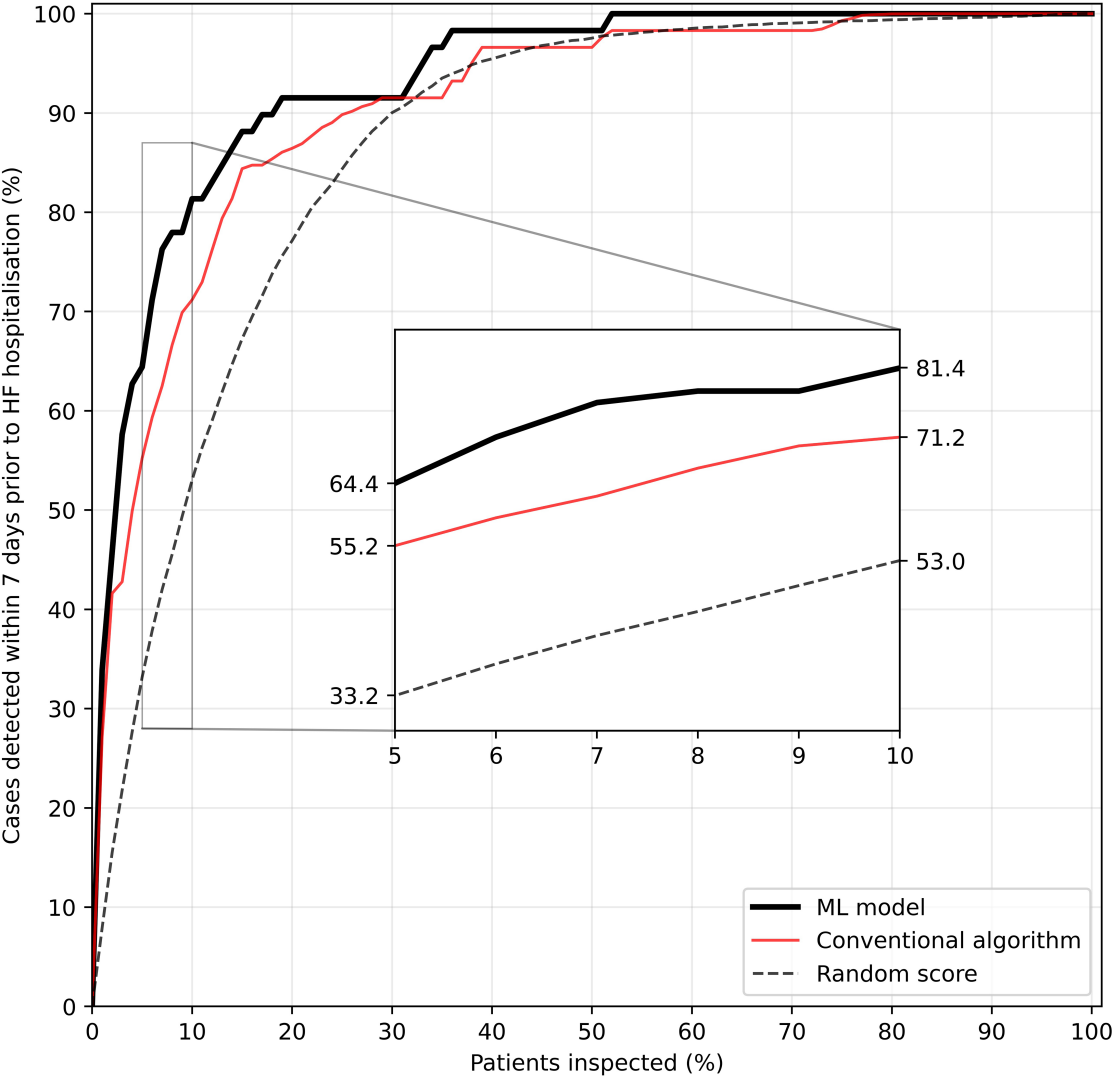
Fraction of HF hospitalisations detected within seven days prior to the event as a function of the fraction of top-ranked patients inspected per day during a one-year RPM setting. A case is considered detected if a patient is among the top-ranked patients inspected at least once in the seven days preceding hospitalisation. Repeated simulations (*n* = 100) were used for tie-breaking in the heuristic rules and for calculating a random score's (randomly allocated number between 0 and 1 per patient and day) performance.

In an RPM scenario where on each day only a fraction of patients would have received medical attention based on highest estimated risk, the ML model performed better than the conventional algorithm with regard to detection of imminent unplanned HF hospitalisations (Figure 5). In this simulation, a case was considered as detected if a particular patient belonged to the fraction of top-ranked patients and would have thus received medical attention at least once in the seven days preceding the event.

The superiority of the ML-based risk score becomes especially obvious when the fraction of inspected top-ranked patients is low. If prioritisation would have been made on the basis on the ML model, evaluating only the 10% highest ranked patients would have led to detection of 81.4% of unplanned HF hospitalisations within the following seven days, an increase of 10 percentage points over the conventional algorithm. To detect 95% of all cases of imminent HF hospitalisations, only the top-ranked one third of all patients would have had to be clinically evaluated on a daily basis in this simulated scenario.

## Discussion

Telemedical HF care has been transitioning from clinical trials to real-life settings. While retrospective studies on its implementation highlight the safety and benefits (37–40), its full potential is far from unleashed (41). For the ongoing implementation—driven by the ESC guidelines (42) and accelerated by the COVID-19 pandemic (41, 43)—the upscaling of capacities is a key issue for providers and patients. In this study, we demonstrated a path towards optimising the operational effectiveness of RPM in HF through artificial intelligence.

We developed and validated an ML-based risk model that considers both the patient's daily condition based on parameters transmitted through non-invasive monitoring devices, as well as the patient's baseline risk described through parameters collected during out-patient visits. The resulting model predicts unplanned HF hospitalisations within seven days in patients undergoing RPM, and out-performs a conventional rule-based algorithm that had been used during the TIM-HF2 trial for priority ranking (26).

**Figure 6:**
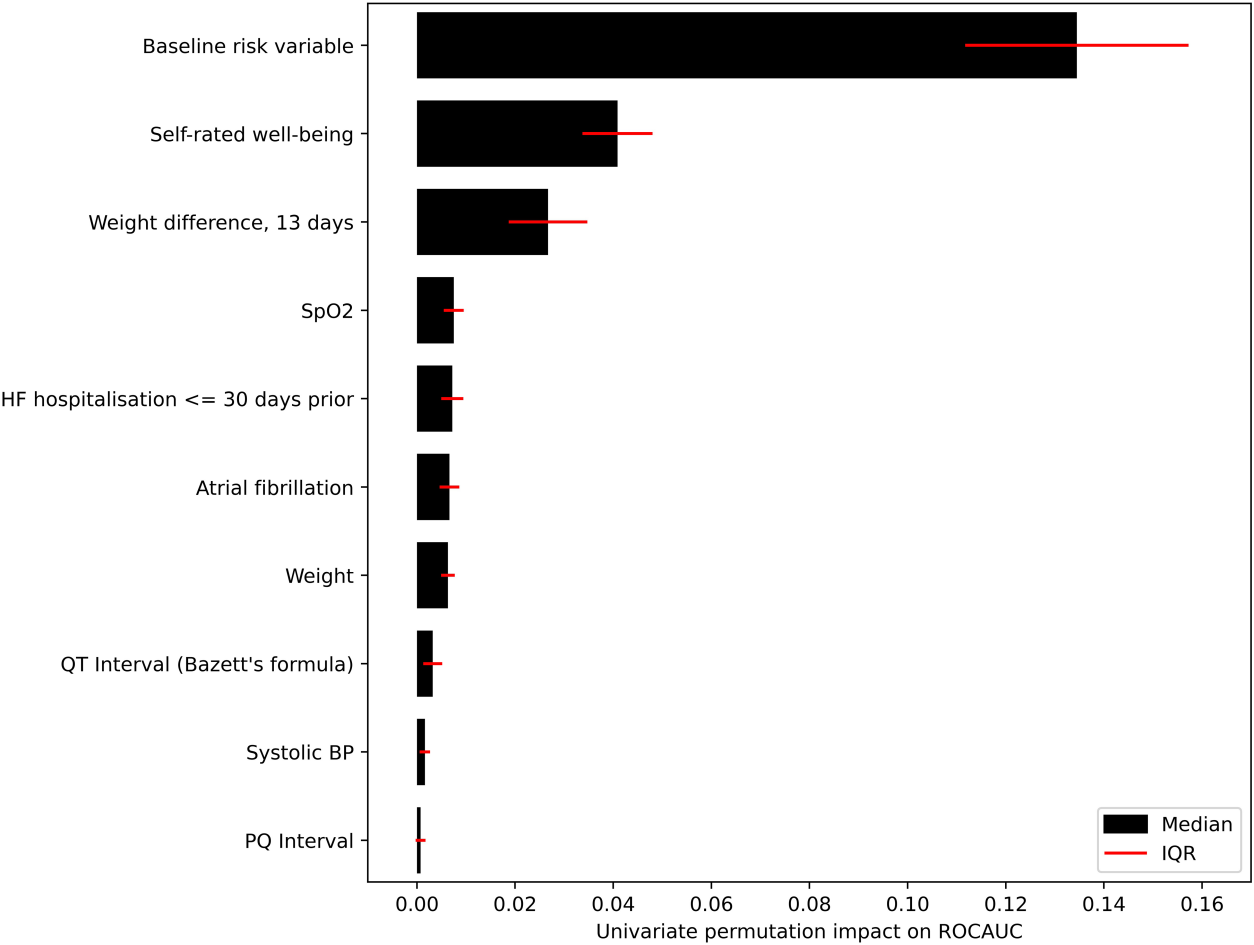
Impact of the 10 highest ranked predictors in the ML model. Impact measured by repeatedly permuting each predictor before making predictions and measuring the resulting drop-off in ROCAUC.

During ML modelling on the daily transmitted data, we faced two key challenges: a severe imbalance between patients with and without HF hospitalisations in the next seven days (training prevalence 0.6%), and a lack of heterogeneity in the training set due to the ∼180,000 data transmissions stemming from only 570 individuals. We sought to alleviate these challenges by using the baseline risk variable as a-priori knowledge of the patient's health condition and passing it as a predictor into the ML model. In the development of the baseline risk variable, we were able to make use of both study arms of the TIM-HF2 trial by explicitly incorporating the information on RPM or usual care (the only structural difference between the cohorts due to the randomized design of the trial) in its development process, which consequently doubled the sample size and increased its robustness. Permutation importance computation confirms that the ML model of unplanned HF hospitalisations relies heavily on this information (Figure 6), and the model achieves high discriminatory performance. This is highlighted both by the ROCAUC of 0.855, and the clear separation between the median scores of patients with and without upcoming HF hospitalisations.

The first key result of this study is the steady upward trajectory of the median ML-based score as patients approach HF hospitalisation starting as early as three weeks prior to the event (Figure 4). This indicates that the model is sensitive to changes in the patients’ health status weeks before acute decompensation, much earlier than the typical onset of acute symptoms like dyspnoea or oedema (44). Sensitivity to changes this early in the HF deterioration process has thus far only been achieved through invasive hemodynamic monitoring (44). In contrast, the developed risk score relies on a single data transmission of multiple vital parameters per day through non-invasive sensors. Thus, through its implementation in the RPM care concept, our findings indicate the potential for timely intervention to reduce the risk of HF hospitalisation.

The second key result is the high detection rate of HF hospitalisation in a simulated one-year RPM scenario, where patients were ranked daily depending on their estimated risk. Ranking based on the ML-based score proved to be especially beneficial when the fraction of top-ranked patients undergoing review is small, and the theoretical gain in patient capacity therefore large. Daily evaluation of the top-ranked 10% of the patient population proved to be sufficient to review over 80% of all cases of HF hospitalisations at least once in the seven days preceding the event, and reviewing the top-ranked third pushes this number to 95% (Figure 5). Thus, the integration of our ML-based risk score in a decision support system (DSS) could fundamentally change the RPM caregiver's workflow by switching from a one-to-one correspondence between data transmission and review to a risk-adjusted review frequency. Reviewing per day a fixed fraction of top-ranked patients could amount to multiplying the patient capacity compared to the daily review of all patients, without additional staff.

In practice, ranking patients solely based on an ML-based score has downsides which need to be accounted for in the implementation of the DSS. No patient, even if classified as stable by the model, should exceed a pre-specified number of days without clinical evaluation. Patients newly added to the program, or recently released from the hospital require special attention to ease the care transition (45), and should be prioritised independent of risk score. Nevertheless, we were able to show that implementing a risk-adjusted review frequency might be a viable approach to increase the operational effectiveness of RPM providers.

This study exhibits three key limitations. First, despite the time series nature of the dataset, the implemented MLP makes little use of the development of predictors over time, except for the inclusion of weight differences. More complex models designed for sequential data could potentially uncover temporal effects like the influence of onsetting or retreating atrial fibrillation. Applying other types of models to this problem, possibly including additional data sources like raw ECG or voice recordings, remains a topic for future studies.

Second, although the TIM-HF2 trial included 765 patients undergoing remote patient management, the validation of our ML-based risk score was performed on a relatively small subset of only 195 patients who were held out from the model development process. This limited validation cohort was necessary to allocate sufficient data for robust training of the ML model. However, the small number of patients in the validation set may limit the generalizability and may increase the susceptibility to outliers. Third, our findings lack external validation. Our findings rely on a retrospective analysis of the TIM-HF2 dataset, including a retrospective simulation approach to estimate fractions of detected cases of HF hospitalisation using the ML-based risk score. A prospective study is needed to test how our proposed approach of reviewing from all daily data transmissions only a pre-selected fraction affects patients’ mortality and morbidity.

## Data Availability

The data analyzed in this study is subject to the following licenses/restrictions: the raw TIM-HF2 trial data cannot be made publicly available, as this is not covered by the written informed consent provided by the participating patients. Limited data access might be obtainable upon reasonable request by contacting the Charité Centre for Cardiovascular Telemedicine or the corresponding author. Requests to access these datasets should be directed to friedrich.koehler@dhzc-charite.de.

## References

[1] Setoguchi S Stevenson LW Schneeweiss S. Repeated hospitalizations predict mortality in the community population with heart failure. Am Heart J. (2007) 154(2):260–6. 10.1016/j.ahj.2007.01.041 17643574

[2] Osenenko KM Kuti E Deighton AM Pimple P Szabo SM. Burden of hospitalization for heart failure in the United States: a systematic literature review. J Manag Care Spec Pharm. (2022) 28(2):157–67. 10.18553/jmcp.2022.28.2.157 35098748 PMC10373049

[3] Lander MM Aldweib N Abraham WT. Wireless hemodynamic monitoring in patients with heart failure. Curr Heart Fail Rep. (2021) 18(1):12–22. 10.1007/s11897-020-00498-4 33420917 PMC7796686

[4] Faragli A Abawi D Quinn C Cvetkovic M Schlabs T Tahirovic E The role of non-invasive devices for the telemonitoring of heart failure patients. Heart Fail Rev. (2021) 26(5):1063–80. 10.1007/s10741-020-09963-7 32338334 PMC8310471

[5] Veenis JF Radhoe SP Hooijmans P Brugts JJ. Remote monitoring in chronic heart failure patients: is non-invasive remote monitoring the way to go? Sensors. (2021) 21(3):887. 10.3390/s21030887 33525556 PMC7865348

[6] Stevenson LW Ross HJ Rathman LD Boehmer JP. Remote monitoring for heart failure management at home. J Am Coll Cardiol. (2023) 81(23):2272–91. 10.1016/j.jacc.2023.04.010 37286258

[7] Koehler F Koehler K Prescher S Kirwan B-A Wegscheider K Vettorazzi E Mortality and morbidity 1 year after stopping a remote patient management intervention: extended follow-up results from the telemedical interventional management in patients with heart failure II (TIM-HF2) randomised trial. Lancet Digit Health. (2020) 2(1):e16–24. 10.1016/S2589-7500(19)30195-5 33328035

[8] Wayne C. L Dariush M David T. L Santosh C. S Stefan D. A Anne B. C The seattle heart failure model. Circulation. (2006) 113(11):1424–33. 10.1161/CIRCULATIONAHA.105.584102 16534009

[9] Ouwerkerk W Voors AA Zwinderman AH. Factors influencing the predictive power of models for predicting mortality and/or heart failure hospitalization in patients with heart failure. JACC Heart Fail. (2014) 2(5):429–36. 10.1016/j.jchf.2014.04.006 25194294

[10] Angraal S Mortazavi BJ Gupta A Khera R Ahmad T Desai NR Machine learning prediction of mortality and hospitalization in heart failure with preserved ejection fraction. JACC Heart Fail. (2020) 8(1):12–21. 10.1016/j.jchf.2019.06.013 31606361

[11] Chicco D Jurman G. Machine learning can predict survival of patients with heart failure from serum creatinine and ejection fraction alone. BMC Med Inform Decis Mak. (2020) 20(1):1–16. 10.1186/s12911-020-1023-5 32013925 PMC6998201

[12] Bradley J Schelbert EB Bonnett LJ Lewis GA Lagan J Orsborne C Predicting hospitalisation for heart failure and death in patients with, or at risk of, heart failure before first hospitalisation: a retrospective model development and external validation study. Lancet Digit Health. (2022) 4(6):e445–54. 10.1016/S2589-7500(22)00045-0 35562273 PMC9130210

[13] Sabouri M Rajabi AB Hajianfar G Gharibi O Mohebi M Avval AH Machine learning based readmission and mortality prediction in heart failure patients. Sci Rep. (2023) 13(1):18671. 10.1038/s41598-023-45925-3 37907666 PMC10618467

[14] Ru B Tan X Liu Y Kannapur K Ramanan D Kessler G Comparison of machine learning algorithms for predicting hospital readmissions and worsening heart failure events in patients with heart failure with reduced ejection fraction: modeling study. JMIR Form Res. (2023) 7:e41775. 10.2196/41775 37067873 PMC10152335

[15] Zheng B Liang T Mei J Shi X Liu X Li S Prediction of 90 day readmission in heart failure with preserved ejection fraction by interpretable machine learning. ESC Heart Fail. (2024). 10.1002/ehf2.15033 PMC11631356 39168476

[16] Averbuch T Sullivan K Sauer A Mamas MA Voors AA Gale CP Applications of artificial intelligence and machine learning in heart failure. Eur Heart J Digit Health. (2022) 3(2):311–22. 10.1093/ehjdh/ztac025 36713018 PMC9707916

[17] Chausiaux OE Keyser M Williams GP Nieznański M Downer PJ Garnett RE Heart failure decompensation alerts in a patient’s home using an automated, AI-driven, point-of-care device. BMJ Case Rep. (2022) 15(4):e248682. 10.1136/bcr-2021-248682 35414581 PMC9006839

[18] Josef S Carsten S Biykem B Jose N-N Peter W Stephan W Continuous wearable monitoring analytics predict heart failure hospitalization. Circ Heart Fail. (2020) 13(3):e006513. 10.1161/CIRCHEARTFAILURE.119.006513 32093506

[19] Gontarska K Wrazen W Beilharz J Schmid R Thamsen L Polze A. Predicting medical interventions from vital parameters: towards a decision support system for remote patient monitoring. In: Tucker A Henriques Abreu P Cardoso J Pereira Rodrigues P Riaño D, editors. Artificial Intelligence in Medicine. Basel, Switzerland: Springer International Publishing (2021). p. 293–7. Available online at: https://link.springer.com/chapter/10.1007/978-3-030-77211-6_33 (Accessed January 11, 2024).

[20] D’Onofrio A Solimene F Calò L Calvi V Viscusi M Melissano D Combining home monitoring temporal trends from implanted defibrillators and baseline patient risk profile to predict heart failure hospitalizations: results from the SELENE HF study. Europace. (2022) 24(2):234–44. 10.1093/europace/euab170 34392336 PMC8824514

[21] Moazeni M Numan L Szymanski MK Van der Kaaij NP Asselbergs FW van Laake LW Monitoring left ventricular assist device parameters to detect flow- and power-impacting complications: a proof of concept. Eur Heart J Digit Health. (2023) 4(6):488–95. 10.1093/ehjdh/ztad062 38045436 PMC10689906

[22] Moazeni M Numan L Brons M Houtgraaf J Rutten FH Oberski DL Developing a personalized remote patient monitoring algorithm: a proof-of-concept in heart failure. Eur Heart J Digit Health. (2023) 4(6):455–63. 10.1093/ehjdh/ztad049 38045433 PMC10689918

[23] Boehmer JP Hariharan R Devecchi FG Smith AL Molon G Capucci A A multisensor algorithm predicts heart failure events in patients with implanted devices: results from the MultiSENSE study. JACC Heart Fail. (2017) 5(3):216–25. 10.1016/j.jchf.2016.12.011 28254128

[24] Cardoso I Cunha PS Laranjo S Grazina A Viegas JM Portugal G Validation of a heart failure risk score in a cohort of cardiac resynchronization therapy patients under remote monitoring: results from the TriageHF^TM^ algorithm. J Innov Card Rhythm Manag. (2023) 14(9):5576–81. 10.19102/icrm.2023.14093 37781719 PMC10540879

[25] Koehler F Koehler K Deckwart O Prescher S Wegscheider K Kirwan B-A Efficacy of telemedical interventional management in patients with heart failure (TIM-HF2): a randomised, controlled, parallel-group, unmasked trial. Lancet. (2018) 392(10152):1047–57. 10.1016/S0140-6736(18)31880-4 30153985

[26] Koehler F Koehler K Deckwart O Prescher S Wegscheider K Winkler S Telemedical interventional management in heart failure II (TIM-HF2), a randomised, controlled trial investigating the impact of telemedicine on unplanned cardiovascular hospitalisations and mortality in heart failure patients: study design and description of the intervention. Eur J Heart Fail. (2018) 20(10):1485–93. 10.1002/ejhf.1300 30230666

[27] Moons KGM Altman DG Reitsma JB Ioannidis JPA Macaskill P Steyerberg EW Transparent reporting of a multivariable prediction model for individual prognosis or diagnosis (TRIPOD): explanation and elaboration. Ann Intern Med. (2015) 162(1):W1–73. 10.7326/M14-0698 25560730

[28] Breiman L. Random forests. Mach Learn. (2001) 45(1):5–32. 10.1023/A:1010933404324

[29] Goodfellow I Bengio Y Courville A. Deep Learning. Cambridge, Massachusetts, USA: MIT Press (2016). p. 800.

[30] Bergstra J Bengio Y. Random search for hyper-parameter optimization. J Mach Learn Res. (2012) 13(10):281–305.

[31] Van Rossum G Drake FL. Python Software Foundation. Available online at: www.python.org (cited July 1, 2021).

[32] Pedregosa F Varoquaux G Gramfort A Michel V Thirion B Grisel O Scikit-learn: machine learning in python. J Mach Learn Res. (2011) 12(null):2825–30.

[33] Abadi M Barham P Chen J Chen Z Davis A Dean J Tensorflow: a system for large-scale machine learning. Proceedings of the 12th USENIX Conference on Operating Systems Design and Implementation. (OSDI’16), USA: USENIX Association (2016), p. 265–83.

[34] Yu M Tham Y-C Rim TH Ting DSW Wong TY Cheng C-Y. Reporting on deep learning algorithms in health care. Lancet Digit Health. (2019) 1(7):e328–9. 10.1016/S2589-7500(19)30132-3 33323206

[35] Robin X Turck N Hainard A Tiberti N Lisacek F Sanchez J-C pROC: an open-source package for R and S+ to analyze and compare ROC curves. BMC Bioinformatics. (2011) 12(1):77. 10.1186/1471-2105-12-77 21414208 PMC3068975

[36] Boyd K Eng KH Page CD. Area under the precision-recall curve: point estimates and confidence intervals. In: Blockeel H Kersting K Nijssen S Železný F, editors. Machine Learning and Knowledge Discovery in Databases. Berlin Heidelberg: Springer (2013), p. 451–66. Available online at: https://link.springer.com/chapter/10.1007/978-3-642-40994-3_29 (Accessed December 12, 2023).

[37] Ploux S Strik M Ramirez FD Buliard S Chauvel R Dos Santos P Remote management of worsening heart failure to avoid hospitalization in a real-world setting. ESC Heart Fail. (2023) 10(6):3637–45. 10.1002/ehf2.14553 37797957 PMC10682851

[38] Auener SL van Dulmen SA Atsma F van der Galiën O Bellersen L van Kimmenade R Characteristics associated with telemonitoring use among patients with chronic heart failure: retrospective cohort study. J Med Internet Res. (2023) 25:e43038. 10.2196/43038 37851505 PMC10620630

[39] Knoll K Rosner S Gross S Dittrich D Lennerz C Trenkwalder T Combined telemonitoring and telecoaching for heart failure improves outcome. NPJ Digit Med. (2023) 6(1):1–9. 10.1038/s41746-023-00942-4 37848681 PMC10582035

[40] Severino P D’Amato A Prosperi S Magnocavallo M Maraone A Notari C Clinical support through telemedicine in heart failure outpatients during the COVID-19 pandemic period: results of a 12-months follow up. J Clin Med Res. (2022) 11(10):2790. 10.3390/jcm11102790 PMC9147859 35628916

[41] Lee KC-S Breznen B Ukhova A Martin SS Koehler F. Virtual healthcare solutions in heart failure: a literature review. Front Cardiovasc Med. (2023) 10:1231000. 10.3389/fcvm.2023.1231000 37745104 PMC10513031

[42] McDonagh TA Metra M Adamo M Gardner RS Baumbach A Böhm M 2021 ESC guidelines for the diagnosis and treatment of acute and chronic heart failure: developed by the task force for the diagnosis and treatment of acute and chronic heart failure of the European Society of Cardiology (ESC) with the special contribution of the heart failure association (HFA) of the ESC. Eur Heart J. (2021) 42(36):3599–726. 10.1093/eurheartj/ehab368 34447992

[43] Xu P Hudnall M Zhao S Raja U Parton J Lewis D. Pandemic-triggered adoption of telehealth in underserved communities: descriptive study of Pre- and postshutdown trends. J Med Internet Res. (2022) 24(7):e38602. 10.2196/38602 35786564 PMC9290332

[44] Angermann CE Rosenkranz S. Telemonitoring und pulmonalisdruck-geführte therapie der herzinsuffizienz. Internist. (2018) 59(10):1041–53. 10.1007/s00108-018-0495-1 30238134

[45] Cowie MR Anker SD Cleland JGF Felker GM Filippatos G Jaarsma T Improving care for patients with acute heart failure: before, during and after hospitalization. ESC Heart Fail. (2014) 1(2):110–45. 10.1002/ehf2.12021 28834628

