# Supplementary material for: Artificial intelligence based real-time prediction of imminent heart failure hospitalisation in patients undergoing non-invasive telemedicine
Source: Front Cardiovasc Med. 2024 Sep 20;11:1457995. doi: 10.3389/fcvm.2024.1457995 (PMC11449733; doi:10.3389/fcvm.2024.1457995)
Supplement: Supplementary file 1 [file Datasheet1.pdf]

Supplementary material

Supplementary Table 1: Limits for numerical base risk predictors. Values exceeding the lower/upper limit were set to missing. If lower/upper limit is not reported, all values on the lower/upper end were left unchanged.

	**Lower limit**	**Upper limit**	**Unit**
**Diastolic blood pressure**	40		mmHg
**Haemoglobin**	4	20	g/dL
**Hematocrit**	15		%
**Leukocytes**		50000	1/µL
**Thrombocytes**	42000		1/µL
**Creatinine**	0.2	8.8	mg/dL
**Sodium**		165	mmol/L
**Potassium**	2	7.5	mmol/L
**NT-proBNP**		35000	pg/mL

Supplementary Table 2: Limits for numerical dynamic risk predictors. Values exceeding the lower/upper limit were set to missing. If lower/upper limit is not reported, all values on the lower/upper end were left unchanged. * Set to missing in case of atrial fibrillation. ** Set to missing if < 0.9x patient's 5-percentile weight or if > 1.1x patient's 95-percentile weight.

	**Lower limit**	**Upper limit**	**Unit**
**Heart rate during ECG**			
Minimum	25	160	1/min
Maximum	40	220	1/min
Average	40	129	1/min
**SpO2**	75		%
**PQ Interval***	50	675	ms
**QRS Interval**	40	290	ms
**QT Interval**	100	780	ms
**QT Interval (Bazett)**	100	780	ms
**Blood pressure**			
Diastolic	40		mmHg
Systolic	70		mmHg
Average	50		mmHg
**Weight****	40		kg

Supplementary Table 3: Heuristic prioritisation rules. Patients are first sorted w.r.t. priority, then w.r.t. points.

**Parameter**	**Rule**	**Priority**	**Points**
Weight	1-day difference > 1 kg	high	1000
	0.5 kg <= 1-day difference <= 1 kg	medium	501
	2-day difference > 2 kg	high	1000
	1.5kg <= 2-day difference <= 2 kg	normal	501
	8-day difference > 2.5 kg	critical	5000
	2 kg <= 8-day difference <= 2.5 kg	medium	501
Self-rated well-being	5 (very bad)	critical	5000
	4 (bad)	high	1000
	3 (normal)	medium	501
	Drop from 1 (very good) to 3 (normal)	medium	501
Blood pressure	Systolic BP < 80 mmHg or<br>Systolic BP > 160 mmHg or<br>Diastolic BP > 100 mmHg	critical	5000
	Systolic BP < 90 mmHg or<br>Systolic BP > 140 mmHg or<br>Diastolic BP > 90 mmHg or<br>Diastolic BP < 40 mmHg	high	1000
	130 mmHg < Systolic BP <= 140 mmHg	medium	501
Ventricular tachycardia	Any occurrence	critical	20000
Atrial fibrillation	First occurrence	critical	10000
	Any other occurrence	medium	501
Sinus rhythm	Any occurrence	low	250
Pacemaker rhythm	Any occurrence	low	250
QRS Interval	First occurrence of<br>QRS Interval < 120 ms (without pacemaker)	critical	5000
	Any other occurrence of<br>QRS Interval < 120 ms (without pacemaker)	low	250

**Parameter**	**Rule**	**Priority**	**Points**
AV block	None	low	250
	I	normal	501
	II	high	1000
	III	critical	5000
Heart rate	Minimal heart rate during ECG<br>< 50 beats per minute or<br>> 100 beats per minute	critical	5000
	Absolute 1-day difference in mean heart rate during ECG<br>> 20 beats per minute	high	1000
QT Interval (Bazett)	>= 460 ms (without pacemaker)	high	1000
	>= 480 ms (without pacemaker)	critical	5000
	1-day relative difference > 10 %	high	1000
SpO2	< 94 %	high	1000
	< 90 %	critical	5000

Supplementary Table 4: Complete list of predictors included in the final models for the baseline risk and the dynamic risk.

	**Predictors**
**Baseline risk**	NT-proBNP
	MR-proADM
	GFR
	Weight
	Heart valve disease
	Current or former smoker
	Procalcitonin
	MR-proANP
	NYHA class III
	Dyspnea on exertion
	COPD
	Leukocytes
	Dilated cardiomyopathy
	Age
	Hematocrit
	HF hospitalisation <= 30 days prior
	Haemoglobin
	Peripheral artery disease
	Diastolic BP
	Living in rural area
	Body-mass index
	Treated with antiplatelet therapy
	Sodium
	Treated with β-blockers
	Stroke
	Fluid intake <= 1.5 L/day

	**Predictors**
	LVEF
**Unplanned HF hospitalisation within 7 days**	Base risk score
	Self-rated well-being
	Weight difference, 13 days
	SpO2
	HF hospitalisation <= 30 days prior
	Atrial fibrillation
	Weight
	QT Interval (Bazett's formula)
	Systolic BP
	PQ Interval
	Average heart rate during ECG
	Minimal heart rate during ECG
	AV block I
	Maximal heart rate during ECG

Supplementary Figure 1: Impact of the 10 highest ranked predictors of the baseline risk. Impact measured by repeatedly permuting each predictor before making predictions and measuring the resulting drop-off in ROCAUC**.**

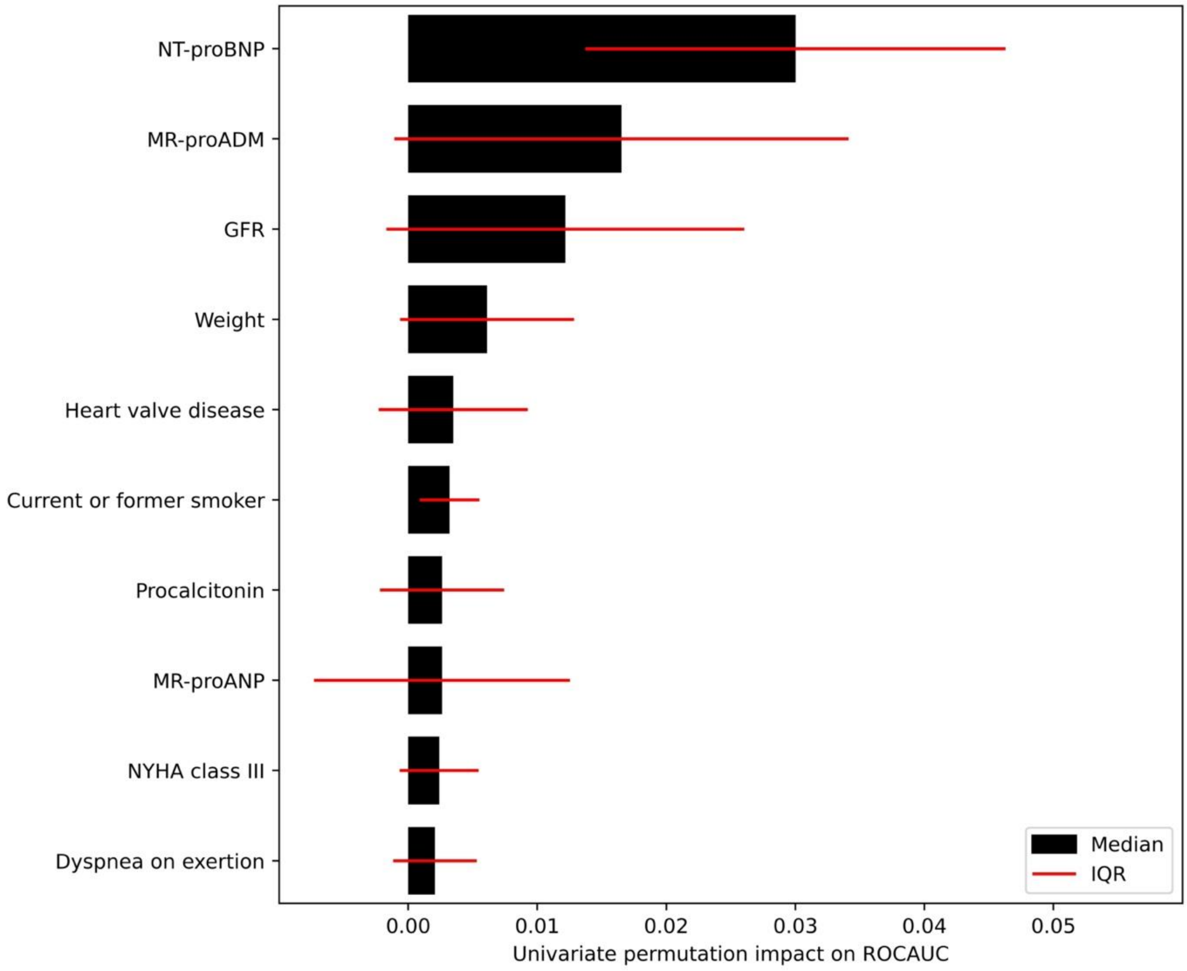

S1: Random Forest implementation details

We train a Random Forest of 250 trees without a pre-specified maximum tree-depth. Tuneable hyperparameters are the maximum number of features per tree (between 3 and 8), the minimum number of samples required in a tree-leaf (between 2 and 15), the minimum number of samples required to allow a further tree-split (between 5 and 30), and the fraction of samples used per tree (evenly spaced between 0.7 and 1).

We use 10-fold cross-validation and 50 random draws from the hyperparameter space in the tuning process.

With the optimal choice of hyperparameters, we compute the permutation importance of all predictors. In cross-validation fashion (10 folds), a model is fit on the training folds using all features, and predictions are made on the test fold with each predictor in turn being randomly permuted. Per fold, we repeat the random permutation of each predictor 25 times. The resulting importance score is the median dropoff in ROCAUC (compared with the ROCAUC without permutation) across all folds and permutation-repeats.

We use the permutation importance scores for backwards predictor elimination. Predictors are sorted by their importance score. In ten evenly spaced steps, we eliminate the lowest ranked predictors until only 10 % of all predictors remain. With all ten tested combinations of features, we run hyperparameter tuning, and select the combination of predictors yielding the largest cross-validated ROCAUC.

S2: Multilayer Perceptron implementation details

The implemented Multilayer Perceptron is trained for 25 epochs with a batch size of 1000. The activation function in all but the last layers is the rectified linear unit (ReLU). We use binary crossentropy as the loss function, and assign each observation a weight inversely proportional to its class frequency. We tune the number and size of hidden layers (one to three layers, 100 to 500 neurons per layer, the sum across all layers not exceeding 500), an L1 and L2 penalty (evenly spaced on the log-scale between 10e-5 and 10e-3), a dropout fraction (evenly spaced between 0 and 0.3), and the learning rate (evenly spaced on the log-scale between 10e-5 and 10e-3).

To model the effect of weight changes on the risk of unplanned HF hospitalisation, the optimal combination of three to 15-day weight differences is chosen via cross-validation during hyperparameter optimization.n We add either one or two weight differences between 2 and 15 days. In case of two differences, the smaller is restricted to be less or equal to four, and the two parameters need to be at least 7 apart.

Hyperparameter tuning consists of 50 random draws from the hyperparameter space and 5-fold cross-validation.

Using the optimal choice of hyperparameters, we compute the permutation importance scores per predictor in the same fashion as described above for the Random Forest model (using 5-fold, instead of 10-fold cross-validation). For the final model, we select only predictors with a permutation importance larger than zero, and re-do the hyperparameter tuning with only those.

S3: Merging the baseline risk variable and the model for daily assessment of risk of HF hospitalisation

To make the output of the baseline risk model available for the daily risk calculation without data leakage, we assigned patients from the training set a base risk score using leave-one-out predictions. While the base risk model was trained only on predictors collected during the baseline examination, predictions used in the risk model for unplanned HF hospitalisation were updated based on the latest available information collected during later quarterly examinations performed as part of the trial.
